# On Fractures of the Thigh and Leg

**Published:** 1833

**Authors:** 

**Affiliations:** Baltimore


ART. V.—*Fractures of the Thigh and Leg.*—We have received in exchange, the first number of a new quarterly periodical, entitled the *Baltimore Medical and Surgical Journal and Review*, edited by Professor Geddings, of the University of Maryland. It is handsomely gotten up, and contains a number of able articles; from the first of which, by Professor Smith, we shall make such extracts as will put our readers in possession of the professor's views on the subject announced at the head of this paragraph. In the following propositions our author sets forth the indications to be fulfilled in the treatment of fractures of the thigh and leg:

1. The leading indication in the treatment of fractures of the lower extremity, appears to me to be, to furnish such mechanical support for the injured member as shall, in as great a degree as possible, act as a substitute for the bone which has suffered fracture. To do this, in regard to those indispensable offices which the bone performs when the body is at rest, is, in a great degree, practicable, as we shall presently see.

2. The next great desideratum is, to furnish such a support or bed for the member, as shall diffuse the pressure, which the weight of the limb occasions, equally over the whole inferior semi-circumference of the leg and thigh. This indication is but very imperfectly accomplished by any apparatus hitherto described.

3. Another not less important object should be, to render the two former indications consistent with a change of posture in the patient's body, and, as far as possible, with freedom of motion in all other parts, the apparatus being an appendage of the body rather than of the bed, and moving with it in all unavoidable motions.

4. It is not less important to place the member in that attitude which shall most perfectly relax the muscles that operate on the fragments of the broken bone.

5. The apparatus should be such as to allow the attitude of the member to be occasionally varied, without the necessity of taking away the supports of the fractured bone.

6. The supports of the limb should be such that, by the removal of some of its appendages, the surgeon, in compound fractures especially, may expose, examine the member, and, if necessary, renew dressings, without at all disturbing its posture or principal supports; in short, without allowing any motion of the fragments.

7. Permanent extension, for the purpose of counteracting the contraction of muscles, and obviating the imperfections of inadequate apparatus, is, in any efficient degree, absolutely impossible; and if all the indications named above be satisfactorily accomplished, is altogether unnecessary.

On each of these propositions the professor has an extended commentary, which we shall pass over, for the purpose of presenting our readers with the description of an apparatus, and its mode of application, which he thinks better calculated than any other, to accomplish the various objects in view.

*Description of the Apparatus.*—The construction of the apparatus will be more intelligible, if it be stated in the first place, that it consists of four pieces, viz. two concave inclined planes, the one adapted to the inferior surface of the thigh—the other to that of the leg. These are united by a hinge corresponding to the knee. The third piece is for the foot, and the fourth is connected with the thigh piece and extends upward beside the body, and this we will call the hip piece.

The thigh piece is constructed thus. Two narrow pieces of strong oak, or other hard wood, are to be prepared, twelve and a half inches long, half an inch thick, and seven-eighths of an inch wide. A piece of very thick strong sheet iron, fifteen inches long and an inch wide, is next to be taken, and at each end it must be bent at right angles, first an inch from the end, then again an inch from this, and finally an inch from this last, so that at each end a socket is formed for the end of the piece of wood, an inch square, thus. 

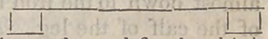

This socket is large enough to receive the piece of wood for the thigh and also the hip piece. Four holes are made in the sides of the socket by which to screw it to the wood. The piece of iron is now to be bent in the middle, so as to be adapted to the shape of the thigh, and must be bent in a direction opposite to that of the ends for forming the sockets. It should be bent more in the middle than the sides, so that it shall be deeper than a semicircle. Its span will be seven inches.—The two pieces of wood are now to have their upper ends placed in the sockets, and screwed tightly to them, leaving the outer part of the socket unoccupied, to slip in the hip-piece, as occasion may require. A semicircular bow of strong wire, with the ends flattened and perforated with holes for screws, is next to be screwed to the pieces of wood about two inches from their inferior ends. Its extremities are most conveniently screwed to the wood pieces on the outside. The span of this wire is six inches. Thus is formed the skeleton of the thigh piece. The concave floor is to be formed of strong cotton duck, (which I have found after several trials to be the best material) a broad piece of which is to be nailed on each side along the upper edges of the pieces of wood, and allowed to fall loosely between them so as almost to reach the concavity of the two irons. It should be deeper at the upper than at the lower part, to suit the tapering form of the thigh. It must be wrapped over the superior edge of the upper iron, (a piece of blanket being interposed to make the margin large and soft) and sewed to itself at the inferior margin of the iron. Thus we have an easy, firm and well adapted surface for the thigh to rest upon. To secure the thigh in the splint, take a piece of poplar board one-tenth of an inch thick, twelve inches long, and six wide; glue a piece of strong domestic cotton to one of its surfaces, then split the wood in strips half an inch wide which will of course be held together by the cloth, but allowed to fold upon each other. Two leather straps are to be tacked across this, and by means of four buckles (two on each side attached to the side of the thigh pieces with pieces of leather and tacks) it is fastened to each side, and may be made to embrace the thigh with any degree of tightness. This is far better than a bandage, because making a more firm, steady and unvarying pressure.

Next we form the leg piece, of two pieces of wood of the same size as those of the thigh, but nineteen and a half inches long. They are permanently fixed parallel to each other, and five and a half inches apart, by means of two bows of strong iron wire, passing beneath and screwed firmly to the outside of each piece, the one about two inches from the superior, the other about four inches from the inferior, extremity of the pieces of wood. Thus the frame of the leg portion is formed. To form the concave floor to receive the calf of the leg, take a piece of cotton duck, about two and a half inches wide and seven inches long; nail the opposite extremities of this to the opposite pieces of wood, the superior margin being about an inch from their upper extremities. Let the middle portion sink loosely like a festoon, between the pieces, so that when the leg rests on it, it shall be pressed almost down to the iron bow. This cloth will receive the upper part of the calf of the leg. Another similar piece is to be attached about three inches from the inferior extremities, but as this receives the ankle, it must not sink so deeply between the pieces. These are the only permanent pieces of cloth which are used for this purpose; others are to be placed along the splint at the moment of applying it to the limb, and fitted to the calf of the leg all along, being fastened at the time with tacks to the side pieces, and accurately adjusted to the form of the limb by drawing the ends over the side pieces, until they are found to support their portion of the weight of the limb. By these the pressure of the weight of the limb is equally diffused.

The thigh and leg portions are now to be united by means of two hinges, one on each side. Each of these is formed of two pieces of very strong sheet iron, as wide as the pieces of ash used in the splint. The upper one, to be attached to the thigh piece, is five inches long. Two inches of its length are screwed to the outside of the thigh piece, and, of course, it projects three inches beyond it. An oblong slit is made in this, commencing at the end of the piece of wood and extending almost to the end of the iron, the slit being about two inches and a half long and a quarter of an inch wide. The end of this iron may be filed semi-circularly. The lower piece of the hinge is to be two inches and a quarter long. It is screwed in the same manner to the leg splint, and extends beyond the piece of wood only three quarters of an inch. In the centre of this projecting piece is a round hole, equal in diameter to the oblong hole in the other piece. These two pieces are now to be joined together with a thumb screw, the head of which is on the inside, and the screw with the thumb piece, external. This hinge will allow of free flexion and extension, and as the screw may glide along the oblong slit, we may at pleasure elongate or abbreviate the thigh piece two and a half inches, which is as much as the thighs of different persons differ in length. When the thumb screws are tight, motion is checked; but something is necessary, when the splint supports the weight of the limb, to make it perfectly inflexible to any angle that may be desired. This is accomplished by attaching a narrow piece of sheet iron, four inches long, to the centre of the iron bow which fastens together the thigh pieces, by merely wrapping one of its ends round it, so as to form a sort of hinge. This piece is to have an oblong slit in it, beginning near the iron bow, and extending near to its extremity; it must also be bent so as to be convex upward. A hole should be made in the centre of the upper bow of the leg portion; then a thumb screw is put through this and through the narrow iron which (when the thigh and leg portions are bent upon each other) comes beneath it. When the screw is not close, the narrow iron will easily slide along the screw, and allow any angle to be formed, and the moment that it is tightly turned, the angle is permanently fixed.

The foot piece is formed of a half inch board cut in the shape of the sole of a shoe. Around the margin of the heel a piece of firm leather is nailed, projecting about two and a half inches, and designed to support the heel. An oblong slit is made in this piece of wood, beginning about an inch from the heel, and extending longitudinally about two and a half inches towards the toe. This is attached to the lower extremities of the leg portion, by means of a piece of sheet iron, as wide as the pieces of wood in the splint, and bent twice at right angles, so that when the side pieces of it are applied to the sides of the leg portion, they will overlap them externally, and the middle part will extend across the extremity from one piece of wood to the other. Each of the side portions of this iron has an oblong slit two and a half inches long, and there is a hole made in the end of each of the pieces of wood. By these holes the iron is attached with a thumb screw, and the oblong slit will slide up and down two inches or more, allowing us to elongate the leg piece in that degree. As they form a sort of hinge, the lower part of it may be elevated or depressed, thus giving any desirable inclination to the foot, which, as will be presently seen, is supported by this piece. A hole being made in the centre of the middle cross portion of this iron, the foot piece of wood, is to be attached to its inside with a thumb screw, passed through the oblong slit, the thumb portion being below. The foot piece will then glide up and down, and may be in a moment adjusted to receive any part of the weight of the foot and leg on the leather heel; or the toe may be turned out or in, to suit the right or left limb, and to give any desired inclination to the foot.

The hip piece is of ash or oak, nine inches long, a little convex externally, and concave internally, to suit the form of the hip. One extremity is to be rounded and fitted into the socket of the thigh splint, and it is to be fastened with a screw, so as to give it some degree of hinge-like motion. Its inner side is to have a thick compress of soft cloth nailed to it, and somewhat fashioned to the shape of the hip. The superior extremity extends to the lowest rib, or higher. This is secured to the body by a piece of strong cotton webbing, two yards long. To one end of this a buckle is attached, and the band is nailed to the outside of the hip piece, at right angles, so that the buckle shall be only a few inches from the piece and in front of the body. The other long tail of the band is now to be carried down behind the haunch—up along the perineum, between the thighs so as to embrace the thigh part of the apparatus at its upper part—then spirally up over the groin to the outer side of the hip, over the hip piece and then around the back—the left loin, and so completely round the body to the buckle, where it is to be secured. This I have found far more permanent and secure than the bandage.

The application of the apparatus may be thus effected. When the fracture is one of the thigh, it is applied entire. The surgeon causes the limb to be raised by assistants, the leg being bent at an easy angle on the thigh, and the splint bent to correspond. The apparatus is then brought under the limb by the surgeon, and the assistants let the member sink into the hollow for its reception, care being taken to press the upper margin of the thigh piece snugly against the perineum and the tuber of the ischium, against which it should rest. The surgeon then grasps the knee, and extending the thigh, coaptates the fragments. The leg is now laid into the corresponding portion, the foot being received into the foot piece, and the upper part of the calf and the ankle resting on the permanent slings. Other strips are now to be drawn under the calf, so as to receive their portion of weight, and are tacked to the side pieces. The anterior short splint is next to be buckled snugly over the thigh, a thick compress being first applied, and compresses being placed between the sides of the splint and the receding parts of the thigh and knee. The hip band is now to be buckled. Next, the surgeon takes a roller and beginning its application at the foot, at first binds the toe to the foot piece; then, he carries it spirally around the heel and splint, and brings it back again to the top of the foot around which it once more passes and then ascends upon the leg, making repeated turns as high as the knee, compresses being used to fill up all inequalities between the sides of the splint and the leg. Here it will be observed that each turn of the bandage gives new support to the leg beneath, and adapts the surface of the support more accurately. Should the length of either thigh or leg piece not correspond to that of the member, it may be adjusted in a moment, as heretofore described. The ankle may also be varied at pleasure.

The whole is then to be elevated from the bed, and suspended as I have already described. The hooks which hold the apparatus should be attached below the knee. At the Baltimore Infirmary, I use a matress which has an oblong portion in the angle, corresponding to the leg piece, cut out, so that the foot swings clear without being raised very high. This also causes the limb by its gravity to exercise some degree of traction on the muscles, and to assist in preventing the shortening of the limb.

When the fracture is one of the leg near the knee, the hip piece may be removed, but the thigh piece must be retained; first, because it more effectually prevents motion at the place of fracture, and causes the splint to move with the body, and next because no more perfect support for the thigh can be devised, even if it were of no other use than for this purpose. When, however, the fracture of the leg is near the ankle, the thigh piece may be dispensed with. The leg piece must then be adjusted with great care, and particular attention must be given to the foot piece, for the proper support of the foot is the principal source of difficulty in these fractures. But with this apparatus we have complete command of it. We can fix the foot in a moment so as to be perfectly immovable, and can with great ease render its attitude precisely such as we desire. The toe should be a little inclined forward and outward, and the pressure should be carefully equalized between the ankle and the heel by elevating or depressing the foot piece. Deformity in these fractures, often results from the sinking of the heel. This we can with ease prevent without causing it to suffer from pressure, for the foot is partly supported on the wooden sole, and the leather for the heel is concave. Short splints of binders' board may be applied to the calf or shin if desired.

It will now be apparent, that, in dressing the injured member, there is no necessity for disturbing the supports in the least. All the bandages may be removed and the limb viewed as it lies in the hollow of the apparatus. If any of the slings are not sufficiently tight, they can be drawn up and adjusted. If it be a compound fracture, the wound can be dressed with perfect facility, and by placing a tin trough beneath it to conduct the water into a bucket, as we practice in the Baltimore Infirmary, a thorough ablution may be formed without disturbing even its posture.

It will be observed that I have not spoken of the many-tailed bandage. The apparatus above described, embraces the member so snugly, that I deem it altogether unnecessary.

The apparatus is equally applicable to injuries of the knee and ankle joints.

